# Belief Propagation Algorithm for Portfolio Optimization Problems

**DOI:** 10.1371/journal.pone.0134968

**Published:** 2015-08-25

**Authors:** Takashi Shinzato, Muneki Yasuda

**Affiliations:** 1 Mori Arinori Center for Higher Education and Global Mobility, Hitotsubashi University, Kunitachi, Tokyo, Japan; 2 Graduate School of Science and Engineering, Yamagata University, Yonezawa, Yamagata, Japan; Southwest University, CHINA

## Abstract

The typical behavior of optimal solutions to portfolio optimization problems with absolute deviation and expected shortfall models using replica analysis was pioneeringly estimated by S. Ciliberti et al. [Eur. Phys. B. **57**, 175 (2007)]; however, they have not yet developed an approximate derivation method for finding the optimal portfolio with respect to a given return set. In this study, an approximation algorithm based on belief propagation for the portfolio optimization problem is presented using the Bethe free energy formalism, and the consistency of the numerical experimental results of the proposed algorithm with those of replica analysis is confirmed. Furthermore, the conjecture of H. Konno and H. Yamazaki, that the optimal solutions with the absolute deviation model and with the mean-variance model have the same typical behavior, is verified using replica analysis and the belief propagation algorithm.

## Introduction

Portfolio optimization is one of the most fundamental frameworks of risk diversification management. Its theory was introduced by Markowitz in 1959 and is one of the most important areas being actively investigated in financial engineering [1–3]. In their theoretical research, Ciliberti and Mézard assessed the typical behavior of optimal solutions to portfolio optimization problems, in particular those described by the absolute deviation and expected shortfall models, using replica analysis, one of the most powerful approaches in disordered systems. With this approach, they showed that the phase transitions of these optimal solutions were nontrivial [1]. However, they did not develop an effective algorithm for finding the optimal portfolio with respect to a fixed return set. This requires a rapid algorithm for resolving the optimal portfolio problem with respect to a large enough in-sample set.

As a first step in such a research direction, we propose an algorithm based on belief propagation, which is well-known as one of the most prominent algorithms in probabilistic inference, to resolve the microscopic averages of the optimal solution in a feasible amount of time for a fixed return set. We also confirm whether the numerical experimental results of our novel algorithm are consistent with the ones of replica analysis. Furthermore, the conjecture of Konno and Yamazaki, that if the return at each period is independently and identically drawn from the normal probability distribution [2], the optimal portfolio of the mean-variance model is consistent with that of the absolute deviation model, is supported using replica analysis and belief propagation.

## Model Setting and Proposed Algorithm

Let us define the model setting for our discussion. A portfolio of *N* assets and the return at period *μ* are represented by **w** = {*w*_1_,*w*_2_, ⋯, *w*_*N*_}^T^ ∈ **R** ^*N*^ and **x**_*μ*_ = {*x*_1*μ*_,*x*_2*μ*_, ⋯, *x*_*Nμ*_}^T^ ∈ **R** ^*N*^, respectively, where *w*_*k*_ is the position of asset *k*, and we assume for simplicity that the mean of the return of asset *k* in period *μ*, *x*_*kμ*_, is zero. The notation T indicates matrix transposition. Given a return set for *p* periods as reference, the problem is to minimize the following cost function (i.e., Hamiltonian) for the portfolio:

$$\begin{array}{ccc} H\left(w\right) & = & \sum_{\mu =1}^{p}R\left(\frac{w^{\text{T}}{\text{x}}_{\mu }}{\sqrt{N}}\right), \end{array}$$ (1)

where *R*(*u*) represents a cost function, such as $\frac{u^{2}}{2}$ in the mean-variance model and ∣*u*∣ in the absolute deviation model, respectively. Furthermore, since the budget is assumed to be finite, the following global constraint is set:

$$\begin{array}{ccc} \sum_{k=1}^{N}w_{k} & = & N. \end{array}$$ (2)

One of our aims is to develop an effective general algorithm for solving this problem; in particular, our aim is an algorithm that works for all cost functions *R*(*u*) and all probability distributions of the returns.

As a basis for the proposed algorithm, following examples in statistical mechanics, we set the joint probability of portfolio **w** used in Eq (1) using finite inverse absolute temperature *β* as follows:

$$\begin{array}{ccc} P(w) & \propto & P_{0}\left(w\right)\exp[-\beta H(w)]\\ & \propto & \prod_{\mu =1}^{p}[P_{0}\left(w\right)g(\frac{w^{\text{T}}{\text{x}}_{\mu }}{\sqrt{N}})]P_{0}^{1-p}\left(w\right), \end{array}$$ (3)

where *g*(*u*) = *e* ^−*βR*(*u*)^ is the likelihood function and prior probability $P_{0}(\text{w})\propto \text{exp}\left[\tilde{m}\left(\sum_{k=1}^{N}w_{k}-N\right)\right]$ for sufficiently large *N* [4, 5]. Notice that the partition function of this posterior probability

$$\begin{array}{ccc} Z & = & \sum_{w}\prod_{\mu =1}^{p}[P_{0}\left(w\right)g(\frac{w^{\text{T}}{\text{x}}_{\mu }}{\sqrt{N}})]P_{0}^{1-p}\left(w\right), \end{array}$$ (4)

is implicitly ignored in this analysis because intuitively it is possible to evaluate the first- and second-order moments of portfolio *w*_*k*_ approximately without the partition function by the following procedure. An arbitrary test probability of portfolio is defined as follows:

$$\begin{array}{ccc} Q(w) & \propto & \prod_{\mu =1}^{p}b_{\mu }(w)\prod_{k=1}^{N}b_{k}^{1-p}\left(w_{k}\right), \end{array}$$ (5)

where the reducibility condition on beliefs *b*_*k*_(*w*_*k*_) and *b*_*μ*_(**w**),

$$\begin{array}{ccc} b_{k}\left(w_{k}\right) & = & \sum_{w\backslash w_{k}}b_{\mu }(w), \end{array}$$ (6)

must hold and **w**\*w*_*k*_ denotes a subset of **w** from which *w*_*k*_ is excluded. The Kullback-Liebler divergence (KLD)

$$\begin{array}{ccc} KL(Q|P) & = & \sum_{w}Q\left(w\right)\log\frac{Q(w)}{P(w)} \end{array}$$ (7)

provides a useful guideline for deriving the belief propagation algorithm. However, since it is too complicated to directly assess KLD except in specific graphical models, we here approximate the Bethe free energy denoted as follows:

$$\begin{array}{ccc} F_{\text{Bethe}} & = & \sum_{\mu =1}^{p}\sum_{w}b_{\mu }\left(w\right)\text{log}\left(\frac{b_{\mu }(w)}{P_{0}\left(w\right)g\left(\frac{w^{\text{T}}{\text{x}}_{\mu }}{\sqrt{N}}\right)}\right)\\ & & +\left(1-p\right)\sum_{k=1}^{N}\sum_{w_{k}}b_{k}\left(w_{k}\right)\text{log}\left(\frac{b_{k}\left(w_{k}\right)}{P_{0k}\left(w_{k}\right)}\right), \end{array}$$ (8)

where $P_{0k}(w_{k})\propto e^{\tilde{m}w_{k}}$ is used. The purpose of this step is to derive the optimal portfolio using the beliefs *b*_*k*_(*w*_*k*_) and *b*_*μ*_(**w**) that minimize the Bethe free energy under the reducibility condition of Eq (6). By adding the term $\sum_{\mu =1}^{p}\sum_{k=1}^{N}\sum_{w_{k}}\lambda_{k\mu }(w_{k})\left[\sum_{\text{w}\backslash w_{k}}b_{\mu }(\text{w})-b_{k}(w_{k})\right]$ to the right-hand side of Eq (8), it is possible to minimize the Bethe free energy with respect to the beliefs to obtain

$$b_{k}\left(w_{k}\right)\;\propto \;P_{0k}\left(w_{k}\right)\exp[\frac{1}{1-p}\sum_{\mu =1}^{p}\lambda_{k\mu }\left(w_{k}\right)],$$ (9)

$$b_{\mu }\left(w\right)\;\propto \;P_{0}(w)g(\frac{w^{\text{T}}{\text{x}}_{\mu }}{\sqrt{N}})\exp[-\sum_{k=1}^{N}\lambda_{k\mu }\left(w_{k}\right)].$$ (10)

Furthermore, for simplicity, we set

$$\begin{array}{ccc} {\tilde{\lambda }}_{k\mu }\left(w_{k}\right) & = & \frac{1}{1-p}\sum_{\mu =1}^{p}\lambda_{k\mu }\left(w_{k}\right)+\lambda_{k\mu }\left(w_{k}\right), \end{array}$$ (11)

as novel auxiliary functions, and then *b*_*k*_(*w*_*k*_) and *b*_*μ*_(**w**) can be rewritten using $\frac{1}{1-p}\sum_{\mu =1}^{p}\lambda_{k\mu }(w_{k})=\sum_{\mu =1}^{p}{\tilde{\lambda }}_{k\mu }(w_{k})$ and $\lambda_{k\mu }\left(w_{k}\right)=-\sum_{\nu (w\mu )}{\tilde{\lambda }}_{k\nu }\left(w_{k}\right)$ as

$$b_{k}\left(w_{k}\right)\;\propto \;P_{0k}\left(w_{k}\right)\exp[\sum_{\mu =1}^{p}{\tilde{\lambda }}_{k\mu }\left(w_{k}\right)],$$ (12)

$$b_{\mu }\left(w\right)\;\propto \;P_{0}\left(w\right)g(\frac{w^{\text{T}}{\text{x}}_{\mu }}{\sqrt{N}})\exp[\sum_{k=1}^{N}\sum_{\nu (\neq \mu )}{\tilde{\lambda }}_{k\nu }\left(w_{k}\right)].\;$$ (13)

Moreover, applying the cumulant generating functions

$$\phi_{k}\left(\theta_{k}\right)=\log\sum_{w_{k}}b_{k}\left(w_{k}\right)e^{w_{k}\theta_{k}},$$ (14)

$$\phi_{\mu }(\theta )=\log\sum_{w}b_{\mu }\left(w\right)e^{w^{T}\theta },$$ (15)

the first and second moments of *w*_*k*_ have the compact forms $m_{wk}=\frac{\partial \phi_{k}(\theta_{k})}{\partial \theta_{k}}=\frac{\partial \phi_{\mu }(\theta )}{\partial \theta_{k}}$ and $\chi_{wk}=\frac{\partial^{2}\phi_{k}(\theta_{k})}{\partial \theta_{k}^{2}}=\frac{\partial^{2}\phi_{\mu }(\theta )}{\partial \theta_{k}^{2}}$ at *θ* = {*θ*_1_, ⋯, *θ*_*N*_}^T^ → 0. This allows us to disregard the calculation of the partition function. Then, our proposed algorithm for sufficiently large *N* comprises the following:

$$m_{wk}=\chi_{wk}(h_{wk}+\tilde{m}),$$ (16)

$$h_{wk}=\frac{1}{\sqrt{N}}\sum_{\mu =1}^{p}x_{k\mu }m_{u\mu }+{\tilde{\chi }}_{wk}m_{wk},$$ (17)

$${\tilde{\chi }}_{wk}=\frac{1}{N}\sum_{\mu =1}^{p}x_{k\mu }^{2}\chi_{u\mu },$$ (18)

$$\chi_{wk}=\frac{1}{{\tilde{\chi }}_{wk}},$$ (19)

$$m_{u\mu }=\frac{\partial }{\partial h_{u\mu }}\log\int_{-\infty }^{\infty }Dzg\;(z\sqrt{{\tilde{\chi }}_{u\mu }}+h_{u\mu }),$$ (20)

$$h_{u\mu }=\frac{1}{\sqrt{N}}\sum_{k=1}^{N}x_{k\mu }m_{wk}-{\tilde{\chi }}_{u\mu }m_{u\mu },$$ (21)

$${\tilde{\chi }}_{u\mu }=\frac{1}{N}\sum_{k=1}^{N}x_{k\mu }^{2}\chi_{wk},$$ (22)

$$\chi_{u\mu }=-\frac{\partial^{2}}{\partial h_{u\mu }^{2}}\log\int_{-\infty }^{\infty }Dzg(z\sqrt{{\tilde{\chi }}_{u\mu }}+h_{u\mu }),$$ (23)

where $Dz=\frac{dz}{\sqrt{2\pi }}e^{-\frac{z^{2}}{2}}$ is used. Note that if ${\tilde{\lambda }}_{k\mu }(w_{k})$ is redefined as ${\tilde{\lambda }}_{k\mu }(w_{k})=-\frac{\gamma k\mu }{2}w_{k}^{2}+{\tilde{h}}_{k\mu }w_{k}$, then ${\tilde{\chi }}_{wk}=\sum_{\mu =1}^{p}\gamma_{k\mu }$ and $h_{wk}=\sum_{\mu =1}^{p}{\tilde{h}}_{k\mu }$[4–7]. In addition, ${\tilde{\chi }}_{wk}m_{wk}$ and ${\tilde{\chi }}_{u\mu }m_{u\mu }$ describe the Onsager reaction terms in the literature of spin glass theory (respectively [8, 9]).

Four points should be noticed here. First, the calculation of this procedure is reduced from *O*(*N* ^3^) to *O*(*N* ^2^). For instance, in the case of the mean-variance model, although we are required to calculate the inverse matrix of the correlation matrix of return set *XX* ^T^ ∈ ℳ_*N*×*N*_, where return matrix *X* = {**x**_1_, ⋯, **x**_*p*_} ∈ ℳ_*N*×*p*_, in order to assess the optimal solution rigorously, it is well-known that this calculation is *O*(*N* ^3^). Moreover, fortunately it is found that in the case of the mean-variance model, this algorithm derives the exact optimal solution (see appendix A for details). Second, only Eqs (20) and (23) are dependent on the likelihood function *g*(*u*) = *e* ^−*βR*(*u*)^, and the variables of index *u* are the only model dependent ones. Furthermore, $\tilde{m}$ is determined by Eqs (2) and (16). Third, the randomness of return is not assumed to be sampled from specific distributions. Because it is plausible that the assumption on the Bethe free energy approximation works correctly if the return at each period is asymptotically not correlated with other returns. Lastly, we expect that in the limit as *β* → ∞, the estimate of the portfolio of asset *k*, *m*_*wk*_, asymptotically corresponds to the optimal portfolio with respect to the given return set.

## Numerical Experimental Results

**Fig 1:**
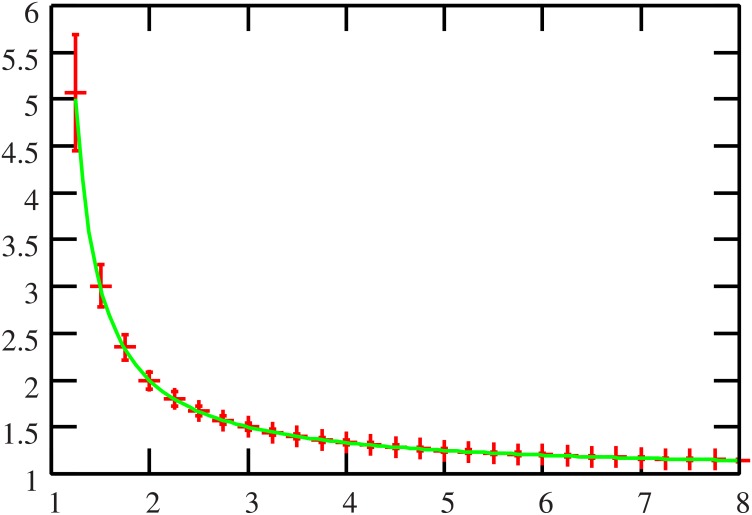
The reference ratio *α* = *p*/*N* (horizontal axis) versus the quenched overlap parameter *q* (vertical axis). The numerical experimental results from the proposed algorithm (error bars) are assessed from 10^2^ experiments using *N* = 500 assets. Comparing with the results of replica analysis (solid line), the effectiveness of proposed algorithm is verified.

**Fig 2:**
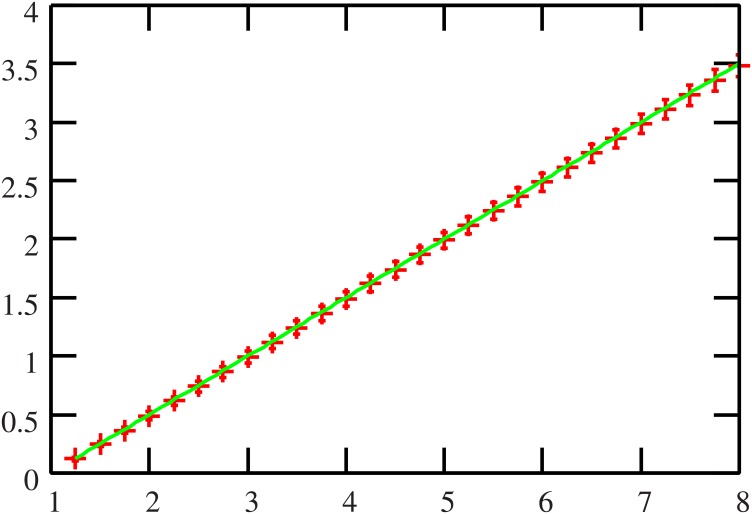
The reference ratio *α* (horizontal axis) versus one degree of the cost function*ε* (vertical axis). This result also indicates that the approximation approach based on probabilistic inference works correctly.

In order to confirm the effectiveness of our method, the numerical experimental results of the proposed algorithm and those of the replica analysis for the case of the Markowitz model are shown in Figs 1 and 2, where *x*_*kμ*_ are independently and identically drawn from the normal distribution with mean and variance 0 and 1, respectively. The numerical experimental result of belief propagation is assessed from 10^2^ samples of the number of assets *N* = 500 and is denoted by error bars and the result of replica analysis is denoted by a solid line. Both findings indicate that the two approaches are consistent with each other.

With regard to the conjecture of Konno and Yamazaki, the variables in Eqs (20) and (23), in the case of the mean-variance model

$$m_{u\mu }=-\frac{\beta }{1+\beta {\tilde{\chi }}_{u\mu }}h_{u\mu },$$ (24)

$$\chi_{u\mu }=\frac{\beta }{1+\beta {\tilde{\chi }}_{u\mu }}$$ (25)

and the absolute deviation model

$$m_{u\mu }=\beta \tanh(\beta h_{u\mu }+\frac{1}{2}\log\frac{H(\beta \sqrt{{\tilde{\chi }}_{u\mu }}+\frac{h_{u\mu }}{\sqrt{{\tilde{\chi }}_{u\mu }}})}{H(\beta \sqrt{{\tilde{\chi }}_{u\mu }}-\frac{h_{u\mu }}{\sqrt{{\tilde{\chi }}_{u\mu }}})}),$$ (26)

$$\chi_{u\mu }=-\frac{\partial m_{u\mu }}{\partial h_{u\mu }},$$ (27)

are assessed exactly using $H(u)=\int_{u}^{\infty }Dz$. Because $H(u)≃{\left(\sqrt{2\pi }u\right)}^{-1}e^{-\frac{u^{2}}{2}}$ in the case of *u* ≫ 1, $m_{u\mu }≃-\frac{h_{u\mu }}{{\tilde{\chi }}_{u\mu }}$ and $\chi_{u\mu }≃\frac{1}{{\tilde{\chi }}_{u\mu }}$ are estimated; that is, this finding indicates that the conjecture of Konno and Yamazaki is valid in part in the sense of the belief propagation approach. See appendices for details.

## Summary

In conclusion, we have discussed an effective algorithm for finding the optimal solution of the portfolio optimization problem with respect to an arbitrary cost function according to Ciliberti and Mézard [1]. With loss of generality, applying the likelihood function *g*(*u*) defined by the cost function *R*(*u*) dependent on the risk diversification problem, we proposed a novel approximation derivation method based on one of the most powerful estimation methods in probabilistic inference. In addition, since two types of Onsager reaction terms are derived in Eqs (17) and (21), our algorithm provides the Thouless, Anderson, and Palmer approach rather than the mean-field approximation in the literature of spin glass theory. One advantage of our algorithm is that it rapidly converges by excluding the effect of self-response. In order to confirm the effectiveness of the proposed approach, we have described the case of the mean-variance model. Furthermore, we have shown that the conjecture of Konno and Yamazaki is supported by employing both approaches developed in cross-disciplinary research involving statistical mechanics and information sciences. In future work, we will assess the properties of *R*(*u*) and the randomness of return that make solving the portfolio optimization problem using belief propagation possible.

## Appendix

### A. Proof of Exactness

We here confirm the exactness of the proposed belief propagation algorithm for the case of the Markowitz model. Our discussion is restricted to *α* > 1 for simplicity. From Eqs (21), (24), and (25), we obtain ${\text{m}}_{u}=-\frac{\beta }{\sqrt{N}}X^{\text{T}}{\text{m}}_{w}$, where **m**_*u*_ = {*m*_*u*1_, ⋯, *m*_*up*_}^T^ ∈ **R** ^*p*^ and **m**_*w*_ = {*m*_*w*1_, ⋯, *m*_*wN*_}^T^ ∈ **R** ^*N*^. Furthermore, $\tilde{m}\text{e}=-\frac{1}{\sqrt{N}}X{\text{m}}_{u}$ follows immediately from Eqs (16), (17), and (19), where **e** = {1, ⋯, 1}^T^ ∈ **R** ^*N*^. Thus, substituting ${\text{m}}_{w}=N\tilde{m}{\left(\beta XX^{\text{T}}\right)}^{-1}\text{e}$ into the constraint *N* = **e** ^T^ **m**_*w*_ gives the exact optimal solution $m_{w}=\frac{N{\left(XX^{\text{T}}\right)}^{-1}\text{e}}{{\text{e}}^{\text{T}}{\left(XX^{\text{T}}\right)}^{-1}\text{e}}$.

### B. Replica Analysis

According to Ciliberti and Mézard, replica symmetry solution of the portfolio optimization problem, where *x*_*kμ*_ is independently and identically distributed with *N*(0,1), is derived as the following extremum:

$$\begin{array}{ccc} -\beta f & = & \lim_{N\to \infty }\frac{1}{N}{\left[\log Z\right]}_{q}\\ & = & \underset{q,\chi }{\text{Extr}}\{\frac{q-1}{2\chi }+\frac{1}{2}\log\chi \\ & & +\alpha \int_{-\infty }^{\infty }Dy\log\int_{-\infty }^{\infty }Dzg\;(z\sqrt{\chi }+y\sqrt{q})\},\; \end{array}$$ (28)

where $Z=\sum_{\text{w}}\;P_{0}(\text{w})\prod_{\mu =1}^{p}g\left(\frac{w^{\text{T}}{\text{x}}_{\mu }}{\sqrt{N}}\right)$ is the partition function and the notation [⋯]_*q*_ denotes the quenched average over the return set. Moreover, the quenched overlap parameters become $q_{ab}=\frac{1}{N}\sum_{k=1}^{N}w_{ka}w_{kb}=\chi +q$ if *a* = *b* and *q* otherwise by employing replica indices *a*,*b* = 1, 2, ⋯, *n* and the assumption of replica symmetry. Furthermore, for large *N* and *p*, *α* = *p*/*N* ∼ *O*(1) remains finite and plays an important role as a control parameter with respect to phase transition phenomena. If $g(u)=e^{-\frac{\beta }{2}u^{2}}$, then $q={\left(1-\frac{1}{\alpha }\right)}^{-1}$ and *χ* = (*β*(*α* − 1))^−1^ can be exactly calculated in the case *α* > 1 and *q* → ∞, and *χ* → ∞ otherwise. This analytical finding is also verified in by the following. It is well known that the eigenvalue distribution of the correlation matrix $C=\frac{1}{N}XX^{\text{T}}$ in the limit of *N* → ∞ is asymptotically close to the Marčhenko-Pastur law $\rho (\lambda )={\left[1-\alpha \right]}^{+}\delta (\lambda )+\frac{\sqrt{{\left[\lambda -\lambda_{-}\right]}^{+}{\left[\lambda_{+}-\lambda \right]}^{+}}}{2\pi \lambda }$ with $\lambda_{\pm }={\left(1\pm \sqrt{\alpha }\right)}^{2}$ and [*u*]^+^ = max{*u*, 0} [10]. Therefore, $q=\langle \frac{1}{\lambda^{2}}\rangle {\langle \frac{1}{\lambda }\rangle }^{-2}$ and one degree of the cost function $\varepsilon ={\text{lim}}_{N\to \infty }\frac{1}{N}{\left[H(w)\right]}_{q}=\frac{1}{2}{\langle \frac{1}{\lambda }\rangle }^{-1}$ are obtained straightforwardly using $\left\langle f(\lambda )\right\rangle =\int_{-\infty }^{\infty }d\lambda \rho (\lambda )f(\lambda )$. Applying Marčhencko-Pastur law, that $\langle \frac{1}{\lambda }\rangle =\frac{\lambda_{+}+\lambda_{-}}{4\sqrt{\lambda_{+}\lambda_{-}}}-\frac{1}{2}=\frac{1}{\alpha -1}$ and $\langle \frac{1}{\lambda^{2}}\rangle =\frac{\sqrt{\lambda_{+}\lambda_{-}}}{2\pi }\times \frac{\pi }{2}{\left(\frac{1}{2}\left(\frac{1}{\lambda_{-}}-\frac{1}{\lambda_{+}}\right)\right)}^{2}=\frac{\alpha }{{\left(\alpha -1\right)}^{3}}$ if *α* > 1 and approach infinity otherwise follows directly. This is consistent with the findings of replica analysis [5].

In general, the order parameters are derived as follows:

$$\chi =-\frac{\sqrt{q}}{\alpha \eta },$$ (29)

$$q=1+\alpha \chi^{2}\delta ,$$ (30)

$$\eta =\int_{-\infty }^{\infty }Dyy\;\left(\frac{\int_{-\infty }^{\infty }Dzg^{\prime }\left(z\sqrt{\chi }+y\sqrt{q}\right)}{\int_{-\infty }^{\infty }Dzg\left(z\sqrt{\chi }+y\sqrt{q}\right)}\right),$$ (31)

$$\delta =\int_{-\infty }^{\infty }Dy\;{\left(\frac{\int_{-\infty }^{\infty }Dzg^{\prime }\left(z\sqrt{\chi }+y\sqrt{q}\right)}{\int_{-\infty }^{\infty }Dzg\left(z\sqrt{\chi }+y\sqrt{q}\right)}\right)}^{2}.$$ (32)

From Eqs (29) and (30),

$${\begin{array}{ccc} q & = & \left(1-\frac{1}{\alpha }\frac{\delta }{\eta^{2}}\right) \end{array}}^{-1}$$ (33)

is obtained. In the limit of sufficiently large *β* of *g*(*u*) = *e* ^−*β*∣*u*∣^, if we assess $\eta ≃-\frac{\sqrt{q}}{\chi }$ and $\delta ≃\frac{q}{\chi^{2}}$ asymptotically, then the conjecture of Konno and Yamazaki is confirmed as correct in the sense of replica analysis.

### C. The Conjecture of Konno and Yamazaki

This conjecture is related to the assessment of an annealed system in the context of spin glass theory. If the return at period *μ*, **x**_*μ*_, is independently and identically drawn from *N* (0, Σ), where Σ ∈ ℳ_*N*×*N*_ is variance-covariance matrix and **w** is fixed, the novel variable $z=\frac{w^{\text{T}}{\text{x}}_{\mu }}{\sqrt{N}}$ is distributed as *N* (0, *s* ^2^(**w**)) with $s^{2}(w)=\frac{1}{N}w^{\text{T}}\sum w\in R$. With respect to fixed **w**, employing one degree of the cost function of the annealed optimization problem $\varepsilon (\text{w})={\left[\frac{1}{N}\sum_{\mu =1}^{p}R\left(\frac{w^{\text{T}}{\text{x}}_{\mu }}{\sqrt{N}}\right)\right]}_{q}=\alpha \int_{-\infty }^{\infty }DuR\left(us(\text{w})\right)$, which becomes $\varepsilon_{\text{MV}}(w)=\frac{\alpha }{2}s^{2}(w)$ in the case of the mean-variance model and $\varepsilon_{\text{AD}}(\text{w})=\frac{2\alpha }{\sqrt{2\pi }}|s(\text{w})|$ in the absolute deviation model. This implies that the optimal portfolios of the annealed situations of the two models are consistent with each other. Note that one degree of the cost function in the case of the annealed portfolio problem with the expected shortfall model, $\varepsilon_{\text{ES}}\left(w\right)=\min_{v\geq 0}\alpha \{v\gamma +H(\frac{v}{s(w)})\}$ with *γ* > 0 can also be assessed. If $s(\text{w})\leq \frac{1}{\sqrt{2\pi }\gamma }$, then this optimal solution is identical to those of the previous mentioned models. This finding, that is, $\arg\min_{w^{T}e=N}\varepsilon_{\text{MV}}\left(w\right)=\arg\min_{w^{T}e=N}\varepsilon_{\text{AD}}\left(w\right)$, is one part of the contributions reported by Konno and Yamazaki.

However, they optimistically assumed **w**_MV_ = **w**_AD_ with respect to a given return set *X* without any mathematical proof, using

$$w_{M\text{V}}=\arg\min_{w^{T}e=N}\frac{1}{2N}\sum_{\mu =1}^{p}\sum_{i=1}^{N}\sum_{k=1}^{N}w_{i}w_{k}x_{i\mu }x_{k\mu },$$ (34)

$$w_{A\text{D}}=\arg\min_{w^{T}e=N}\sum_{\mu =1}^{p}|\frac{1}{\sqrt{N}}\sum_{k=1}^{N}w_{k}x_{k\mu }|.$$ (35)

As explained above, $\arg\min_{w^{T}e=N}\varepsilon_{\text{MV}}\left(w\right)=\arg\min_{w^{T}e=N}\varepsilon_{\text{AD}}\left(w\right)$ with respect to the annealed optimization problem strictly holds; however, **w**_MV_ = **w**_AD_ is not always satisfied. For example, in the simple case of *N* = *p* = 2 for the two returns **x**_1_ = {*a*, *c*}^T^ and **x**_2_ = {*b*, *d*}^T^, their assumption **w**_MV_ = **w**_AD_ does not hold, except under specific special situations.

Although this is apparently contradictory to these obtained findings from both approaches, it is necessary to recognize that the relation **w**_MV_ = **w**_AD_ with a fixed return set is equivalent to the sufficient condition *q*_MV_ = *q*_AD_, where $q_{\text{MV}}={\text{lim}}_{N\to \infty }\frac{1}{N}{\left[{\text{w}}_{\text{MV}}^{\text{T}}{\text{w}}_{\text{MV}}\right]}_{q}$ and $q_{\text{AD}}=\lim_{N\to \infty }\frac{1}{N}{\left[w_{\text{AD}}^{T}w_{\text{AD}}\right]}_{q}$ are quenched averages of overlap parameters. Moreover, although **w**_MV_ = **w**_AD_ does not hold in general, it is expected that the inner product $\frac{w_{\text{MV}}^{\text{T}}w_{\text{AD}}}{\left|w_{\text{MV}}||w_{\text{AD}}\right|}$ is approximately 1 because $\frac{1}{N}\sum_{\mu >v}x_{k\mu }x_{jv}\to 0$ in the case of sufficiently large *N*.

## Supporting Information

S1 Data Figure 1 and figure 2 from S1 Data. S1 Data is the result from numerical experiment of S1 File and S2 File.
(TXT)

S1 File S1 File is main program of C language. (TXT)

S2 File S2 File is sub program of C language. (TXT)

## References

[1] Ciliberti S., Mézard M., 2007 Risk minimization through portfolio replication, The European Physical Journal B., 57(2), 175–180. 10.1140/epjb/e2007-00130-7

[2] Konno H., Yamazaki H., 1991 Mean-absolute deviation portfolio optimization model and its applications to Tokyo stock, Management Science, 37(5), 519–531. 10.1287/mnsc.37.5.519

[3] Markowitz H., 1959 Portfolio selection: Efficient diversification of investments, J. Wiley and Sons, New York.

[4] Kabashima Y., 2005 Replicated Bethe free energy: A variational principle behind survey propagation, Journal of the Physical Society of Japan, 74(8), 2133–2136. 10.1143/JPSJ.74.2133

[5] Shinzato T., 2015 Self-Averaging Property of Minimal Investment Risk of Mean-Variance Model, PLOS ONE. 10.1371/journal.pone.0133846 PMC4520490 26225761

[6] Çakmak B., Winther O., Fleury H. B., 2014 S-AMP: Approximate message passing for general matrix ensembles, IEEE, Information Theory Worksop, 192–196.

[7] Minka, T. P., 2001. Expectation propagation for approximate Bayesian inference, Proceedings of the Seventeenth conference on Uncertainty in artificial intelligence, 362–369.

[8] Opper M., Winther O., 2001 Tractable approximations for probabilistic models: The adaptive Thouless-Anderson-Palmer mean field approach, Physical Review Letters, 86(17), 3695 10.1103/PhysRevLett.86.3695 11329302

[9] Opper M., Winther O., 2001 Adaptive and self-averaging Thouless-Anderson-Palmer mean-field theory for probabilistic modeling, Physical Review E, 64(5), 056131 10.1103/PhysRevE.64.056131 11736038

[10] Shinzato T., Kabashima Y., 2008 Perceptron capacity revisited: Classification ability for correlated patterns, Journal of Physics A, 41(32), 324013 10.1088/1751-8113/41/32/324013

